# Suppression by Ghrelin of *Porphyromonas gingivalis*-Induced Constitutive Nitric Oxide Synthase S-Nitrosylation and Apoptosis in Salivary Gland Acinar Cells

**DOI:** 10.1155/2010/643642

**Published:** 2010-09-01

**Authors:** Bronislaw L. Slomiany, Amalia Slomiany

**Affiliations:** Research Center, C875, University of Medicine and Dentistry of New Jersey Dental School, 110 Bergen Street, P.O. Box 1709, Newark, NJ 07103-2400, USA

## Abstract

Oral mucosal inflammatory responses to periodontopathic bacterium, *P. gingivalis*, and its key virulence factor, LPS, are characterized by a massive rise in epithelial cell apoptosis and the disturbances in NO signaling pathways. Here, we report that the LPS-induced enhancement in rat sublingual salivary gland acinar cell apoptosis and NO generation was associated with the suppression in constitutive nitric oxide synthase (cNOS) activity and a marked increase in the activity of inducible nitric oxide synthase (iNOS). We demonstrate that the detrimental effect of the LPS on cNOS was manifested by the enzyme protein S-nitrosylation, that was susceptible to inhibition by iNOS inhibitor, 1400 W. Further, we show that a peptide hormone, ghrelin, countered the LPS-induced changes in apoptosis and cNOS activity. This effect of ghrelin was reflected in the decrease in cNOS S-nitrosylation and the increase in phosphorylation. Our findings imply that *P. gingivalis*-induced disturbances in the acinar cell NO signaling pathways result from upregulation in iNOS-derived NO that causes cNOS S-nitrosylation that interferes with its activation through phosphorylation. We also show that ghrelin protection against *P. gingivalis*-induced disturbances involves cNOS activation associated with a decrease in its S-nitrosylation and the increase in phosphorylation.

## 1. Introduction

Nitric oxide is a multifunctional, short-lived signaling molecule that plays an important role in a variety of regulatory pathways that are of significance to cellular survival, integrity maintenance, and the modulation of inflammatory responses to bacterial infection [1–3]. The physiological and pathophysiological implications of NO depend on its local concentration, the type of NOS isozyme involved in NO generation, substrate availability, and the enzyme compartmentalization with respect to protein target [1, 3]. Indeed, a low level of NO generated by membrane-associated cNOS appears to access a pool of substrates that are of importance to maintenance of normal physiological functions, including the regulation of apoptogenic signal propagation [3–5]. In contrast, a high level of NO generated by more distant cytosolic iNOS in response to proinflammatory cytokines or bacterial LPS, is of importance to host defense, but its sustained production is associated with the induction of apoptosis [1, 5, 6]. Therefore, the disturbances in cNOS activation associated with bacterial colonization may be a major factor defining the extent of inflammatory involvement.

Studies indicate that cNOS activity is regulated by a complex set of pre- and posttranslational modifications that affect the dynamic of its subcellular targeting and the activity by exposing the enzyme to fatty acid modification through N-myristoylation and thiopalmitoylation, interaction with regulatory cofactors, and the protein phosphorylation with the involvement of Src/Akt pathway [7–10]. Furthermore, there are recent reports that cNOS enzyme protein S-nitrosylation at the critical zinc-tetrathiolate cysteine residues leads to a transient inhibition of cNOS activity [11, 12]. It is also becoming increasingly apparent that, like posttranslational modification through phosphorylation, the protein S-nitrosylation is a targeted and reversible physiologically important posttranslational modification that regulates a large variety of cellular signaling events, including transmittance fidelity of NO signals [3–6, 11]. The S-nitrosylation of mitochondrial protein thiols is known to block cell death after glutathione depletion, and contributes to redox regulatory activity and antiapoptotic function of thioredoxin [3, 13]. More importantly, the cNOS-generated NO has been implicated in the inhibition of apoptogenic signal through S-nitrosylation of the key executioner caspase, caspase-3 [14–16].

Recent advances in identifying the salivary constituents that are capable of influencing the extent of oral mucosal inflammatory responses have brought to focus the importance of a peptide hormone, ghrelin [17]. Emerging evidence indicates that this 28-amino acid peptide, initially isolated from the stomach [18], and more recently identified in saliva and the acinar cells of salivary glands [17], is a principal modulator of the local inflammatory responses to bacterial infection through the regulation of NOS system responsible for NO production [19, 20]. Moreover, we have shown recently that the countering effect of ghrelin on *P. gingivalis *LPS-induced salivary gland cell apoptosis was reflected in the increase in cNOS activity [16].

As the oral mucosal inflammatory responses to periodontopathic bacterium, *P. gingivalis*, are characterized by the enhanced epithelial cell apoptosis and the disturbances in NO signaling pathways [21, 22], in this study we investigated further the nature of the impairment in NOS generating system induced in sublingual salivary gland acinar cells by the LPS of *P. gingivalis. *Our data revealed that the disturbances in the acinar cell NO generation system caused by the LPS results from iNOS-induced suppression of cNOS activation through S-nitrosylation, and that the countering effect of ghrelin was reflected in a marked decrease in cNOS S-nitrosylation and the increase in the enzyme protein phosphorylation.

## 2. Materials and Methods

### 2.1. Salivary Gland Cell Incubation

The acinar cells of sublingual salivary gland, collected from freshly dissected rat salivary glands, were suspended in five volumes of ice-cold Dulbecco's modified (Gibco) Eagle's minimal essential medium (DMEM), supplemented with fungizone (50 *μ*g/mL), penicillin (50 U/mL), streptomycin (50 *μ*g/mL), and 10% fetal calf serum, and gently dispersed by trituration with a syringe, and settled by centrifugation [22]. Following rinsing, the cells were resuspended in the medium to a concentration of 2 × 10^7^ cell/mL, transferred in 1 mL aliquots to DMEM in culture dishes and incubated under 95% O_2_–5% CO_2_ atmosphere at 37°C for 16h in the presence of 0–200 ng/mL of *P. gingivalis *LPS [23]. In the experiments evaluating the effect of ghrelin (rat, Sigma), cNOS inhibitor, L-NAME, iNOS inhibitor, 1400 W, Src inhibitor, PP2, Akt inhibitor, SH-5 (Calbiochem), and ascorbate (Sigma), the cells were first preincubated for 30 minutes with the indicated dose of the agent or vehicle before the addition of the LPS. The viability of cell preparations before and during the experimentation, assessed by Trypan blue dye exclusion assay [23], was greater than 98%.

### 2.2. Porphyromonas Gingivalis Lipopolysaccharide

*P. gingivalis *used for LPS preparation was cultured from clinical isolates obtained from ATCC No. 33277 [23]. The bacterium was homogenized with liquid phenol-chloroform-petroleum ether, centrifuged, and the LPS contained in the supernatant was precipitated with water, washed with 80% phenol solution and dried with ether. The dry residue was dissolved in a small volume of water at 45°C, centrifuged at 100,000 ×g for 4 hours, and the resulting LPS sediment subjected to lyophilization. The preparation was essentially free of nucleic acid and its protein content was less than 0.2%.

### 2.3. Apoptosis and NO Quantification

Quantitative measurement of the acinar cell apoptosis was carried out with a sandwich enzyme immunoassay (Boehringer Mannheim) directed against cytoplasmic histone-associated DNA fragments [22]. The cells from the control and various experimental conditions were settled by centrifugation, rinsed with phosphate-buffered saline, and incubated in the lysis buffer in accordance with the manufacturer's instructions. The lysates were centrifuged at 20,000 g for 10 minutes, and the diluted (1 : 100) supernatant containing the cytoplasmic histone-associated DNA fragments were reacted in the microtiter wells with immobilized antihistone antibody. After washing, the retained complex was reacted with anti-DNA peroxidase, and the immunocomplex-bound peroxidase probed with ABTS reagent for spectrophotometric quantification [22]. To assess NO production in the acinar cells, we measured the stable NO metabolite, nitrite, accumulation in the culture medium using Griess reaction [24]. A 100 *μ*l of spent culture medium was incubated for 10 minutes with 0.1 mL of Griess reagent (Sigma) and the absorbance was measured at 570 nm.

### 2.4. cNOS and iNOS Activity Assay

Nitric oxide synthase activities of cNOS and iNOS enzymes in the acinar cells was measured by monitoring the conversion of L-[^3^H]arginine to L-[^3^H]citrulline using NOS-detect kit (Stratagene). The cells from the control and experimental treatments were homogenized in a sample buffer containing either 10 mM EDTA (for Ca^2+^-independent iNOS) or 6 mM CaCl_2_ (for Ca^2+^-dependent cNOS), and centrifuged [22]. The aliquots of the resulting supernatant were incubated for 30 minutes at 25°C in the presence of 50 *μ*Ci/mL of L-[^3^H]arginine, 10 mM NAPDH, 5 *μ*M tetrahydrobiopterin, and 50 mM Tis-HCl buffer, pH 7.4, in a final volume of 250 *μ*l. Following addition of stop buffer and Dowex-50 W (Na^+^)resin, the mixtures were transferred to spin cups, centrifuged and the formed L-[^3^H] citrulline contained in the flow through was quantified by scintillation counting.

### 2.5. cNOS S-Nitrosylation

Detection of cNOS S-nitrosylation was carried out employing a biotin switch procedure for protein S-nitrosylation [25, 26]. The acinar cells were treated with iNOS inhibitor, 1400 W (30 *μ*M) or ghrelin (0.6 *μ*g/mL) or Src inhibitor, PP2 (20 *μ*M)+ ghrelin and incubated for 16 hours in the presence of 100 ng/mL of *P. gingivalis *LPS. Following centrifugation at 500 ×g for 5 minutes, the recovered cells were lysed in 0.2 mL of HEN lysis buffer (250 mM HEPES, 1 mM EDTA, 0.1 mM neocuproine, pH 7.7), and the unnitrosylated thiol groups were blocked with S-methyl methanethiosulfonate reagent [26]. The proteins were precipitated with acetone, resuspended in 0.2 mL of HEN buffer containing 1% SDS, and subjected to targeted nitrothiol group reduction with sodium ascorbate (100 mM). The free thiols were then labeled with biotin and the biotinylated proteins were recovered on streptavidin beads. The formed streptavidin bead-protein complex was washed with neutralization buffer, and the bound proteins were dissociated from streptavidin beads with 50 *μ*l of elution buffer (20 mM HEPES, 100 mM NaCl, 1 mM EDTA, pH 7.7) containing 1% 2-mercaptoethanol [26]. The obtained proteins were then analyzed by Western blotting.

### 2.6. Western Blot Analysis

The acinar cells from the control and experimental treatments were collected by centrifugation and resuspended for 30 minutes in ice-cold lysis buffer [10]. Following brief sonication, the lysates were centrifuged at 12,000 g for 10 minutes, and the supernatants were subjected to protein determination using BCA protein assay kit (Pierce). The samples, including those subjected to biotin switch procedure, were then resuspended in loading buffer, boiled for 5 minutes, and subjected to SDS-PAGE using 50 *μ*g protein/lane. The separated proteins were transferred onto nitrocellulose membranes, blocked with 5% skim milk, and probed with the antibody against phosphorylated protein at 4°C for 16 hours. After 1 hour incubation with the horseradish peroxidase-conjugated secondary antibody, the phosphorylated proteins were revealed using an enhanced chemiluminescence. Membranes were stripped by incubation in 1M Tris-HCl (pH 6.8), 10% SDS, and 10 mM dithiotreitol for 30 minutes at 55°C, and reprobed with antibody against total cNOS. Immunoblotting was performed using specific antibodies directed against cNOS and phospho-cNOS (Ser^1179^) (Calbiochem).

### 2.7. Data Analysis

All experiments were carried out using duplicate sampling, and the results are expressed as means ± SD. Analysis of variance (ANOVA) followed by nonparametric Kruskal-Wallis test was used to determine significance and the significance level was set at *P* < .05.

## 3. Results

**Figure 1:**
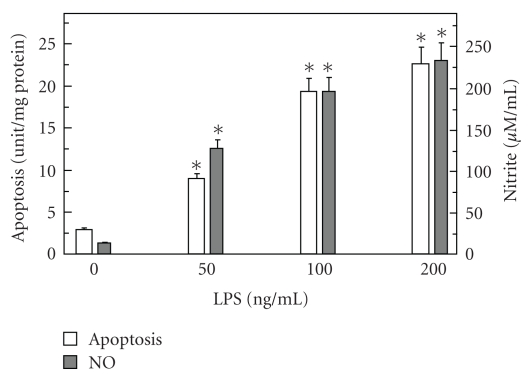
Effect of *P. gingivalis *LPS on rat sublingual salivary gland acinar cell apoptosis and nitrite production. The cells were treated with the indicated concentrations of the LPS and incubated for 16 hours. Values represent the means ± SD of five experiments. *P < .05 compared with that of control (LPS – 0).

**Figure 2:**
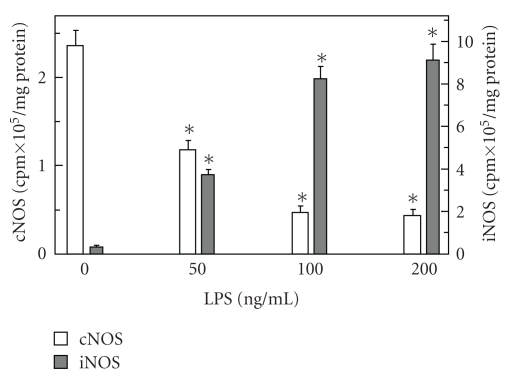
Effect of *P. gingivalis *LPS on the expression of inducible (iNOS) and constitutive (cNOS) nitric oxide synthase activities in rat sublingual salivary gland acinar cells. The cells were treated with the indicated concentrations of the LPS and incubated for 16 hours. Values represent the means ± SD of five experiments. *P < .05 compared with that of control (LPS – 0).

To examine the role of NO generated by cNOS and iNOS isozyme systems in propagation of apoptogenic processes induced by periodontopathic bacterium, *P. gingivalis, *we employed rat sublingual salivary gland acinar cells exposed to the bacterium key virulence factor, LPS. Using apoptotic DNA fragmentation assay in conjunction with the measurements of NO, we demonstrated that *P. gingivalis *LPS caused a dose-dependent increase in the acinar cell apoptosis and NO production, which at 100 ng/mL LPS reached respective values of 6.7- and 15.1-fold over that of controls (Figure 1). We also established that the effect of the LPS at 100 ng/mL on NO production was reflected in a 26.4-fold increase in the acinar cell iNOS activity, while the cNOS activity showed a 5.2-fold decrease (Figure 2).

**Figure 3:**
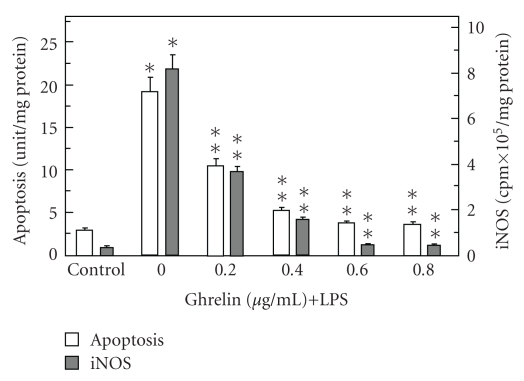
Effect of ghrelin on *P. gingivalis *LPS-induced sublingual salivary gland acinar cell apoptosis and iNOS activity. The cells, preincubated with the indicated concentrations of ghrelin, were treated with the LPS at 100 ng/mL and incubated for 16 hours. Values represent the means ± SD of five experiments. *P < .05 compared with that of control. **P < .05 compared with that of LPS alone.

**Figure 4:**
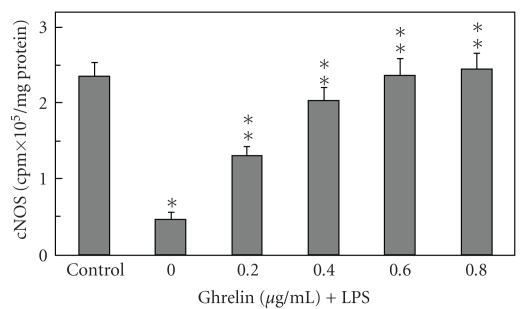
Effect of ghrelin on *P. gingivalis *LPS-induced expression of cNOS activity in sublingual salivary gland acinar cells. The cells, preincubated with the indicated concentrations of ghrelin, were treated with the LPS at 100 ng/mL and incubated for 16 hours. Values represent the means ± SD of five experiments. *P < .05 compared with that of control. **P < .05 compared with that of LPS alone.

Moreover, we found that preincubation with ghrelin led to a concentration-dependent reversal in the LPS-induced changes, which at 0.6 *μ*g/mL resulted in an 80.6% drop in apoptosis as well as a 94.3% decrease in the LPS-induced acinar cell iNOS activity (Figure 3). However, the activity of cNOS in the presence of 0.6 *μ*g/mL ghrelin showed an 80.4% increase over that of the LPS (Figure 4).

**Figure 5:**
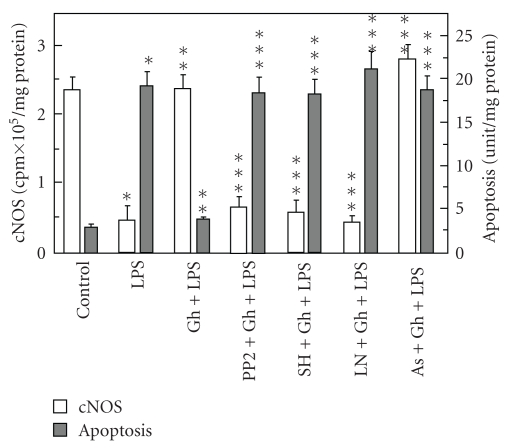
Effect of cNOS inhibitors on the ghrelin (Gh)-induced changes in apoptosis and cNOS activity in the acinar cell exposed to *P. gingivalis *LPS. The cells, preincubated with 20 *μ*M PP2, 30 *μ*M SH-5 (SH), 300 *μ*M ascorbate (As), or 400 *μ*M L-NAME (LN), were treated with Gh (at 0.6 *μ*g/mL) and incubated for 16 hours in the presence of 100 ng/mL LPS. Values represent the means ± SD of five experiments. *P < .05 compared with that of control. **P < .05 compared with that of LPS alone. ***P < .05 compared with that of Gh *+* LPS.

To gain further insight into the relationship between ghrelin-induced upregulation in cNOS activity and the suppression of *P. gingivalis *LPS-induced acinar cell apoptosis, we first focused on examining the effect ghrelin on the events associated with cNOS activation. As cNOS is known to a rapid posttranslational activation through its protein phosphorylation at Ser^1177^ (human) or Ser^1179^ (rat) with the involvement of Src/Akt pathway [9, 10, 20, 27], the acinar cells prior to incubation with ghrelin were pretreated with Src kinase inhibitor, PP2, or Akt inhibitor, Sh-5, or cNOS inhibitor, L-NAME, and assayed for apoptosis. As shown in Figure 5, all three agents significantly inhibited the countering effect of ghrelin on the LPS-induced apoptosis. Moreover, the effects of PP2 and SH-5, like that of L-NAME, were reflected in the inhibition of ghrelin-induced cNOS activity (Figure 5). However, preincubation with nitrosothiols reducing agent, ascorbate [25, 26], produced amplification in the effect of ghrelin on cNOS activity (Figure 5), thus suggesting that in addition to Src/Akt kinase-mediated activation through phosphorylation, the activity of cNOS may be also dependent upon its protein S-nitrosylation.

**Figure 6:**
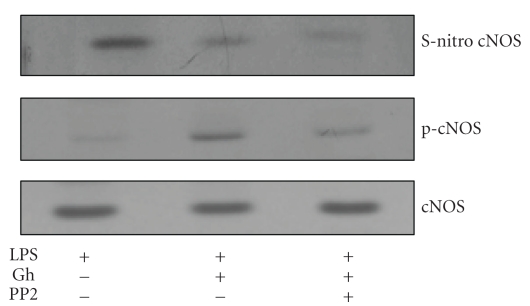
Effect of ghrelin (Gh) on *P. gingivalis *LPS-induced cNOS S-nitrosylation in sublingual salivary gland acinar cells. The cells were treated with Gh (0.6 *μ*g/mL) or PP2 (20 *μ*M) + Gh and incubated for 16 hours in the presence of 100 ng/ml LPS. A portion of the cell lysates was processed by biotin switch method for protein S-nitrosylation and, along with the remainder of the lysates, resolved on SDS-PAGE, transferred to nitrocellulose and probed with phosphorylation-specific cNOS (pcNOS) antibody, and after stripping reprobed with anti-cNOS antibody. The immunoblots shown are representative of three experiments.

To assess further the course of events resulting in the suppression of cNOS activity by *P. gingivalis *LPS as well as to shed a light on the mechanism of ghrelin countering effect, the acinar cells were exposed to incubation with the LPS and ghrelin +LPS, and the lysates subjected to biotin switch procedure were probed with antibodies directed against phospho-cNOS and total cNOS. We observed that the acinar cells exposed to the LPS alone showed a marked increase in cNOS protein S-nitrosylation, while the effect of ghrelin was reflected in the loss in cNOS S-nitrosylation and the increase in its protein phosphorylation (Figure 6). Furthermore, the suppression of ghrelin effect on cNOS activity by Src kinase inhibitor, PP2 (Figure 5), was manifested in the inhibition of cNOS phosphorylation (Figure 6).

**Figure 7:**
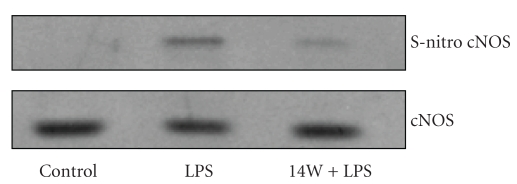
Effect of iNOS inhibitor, 1400 W (14 W) on *P. gingivalis *LPS-induced cNOS S-nitrosylation. The acinar cells were treated with the LPS (100 ng/mL) or 14 W (30 *μ*M) + LPS and incubated for 16 hours in the presence of 100 ng/mL LPS. A portion of the cell lysates was processed by biotin switch procedure for protein S-nitrosylation and, along with the reminder of the lysates, subjected to SDS-PAGE, transferred to nitrocellulose and probed with anti-cNOS antibody. The immunoblots shown are representative of three experiments.

To explore the dependence of cNOS S-nitrosylation on *P. gingivalis* LPS-induced iNOS activity, we pretreated the acinar cells with iNOS inhibitor, 1400 W, and following incubation with the LPS the lysates were subjected to the biotin switch procedure, and examined for cNOS S-nitrosylation. Western blot analysis revealed that the blockage of iNOS activity lead to a substantial decrease in the LPS-induced cNOS S-nitrosylation (Figure 7).

## 4. Discussion

A Gram-negative bacterium, *P. gingivalis*, found in periodontal packets of patients with persistent oral mucosal inflammations, is recognized as a main culprit in the development of periodontal disease that is the major cause of adult tooth loss [28, 29]. The oral mucosal responses to *P. gingivalis *and its key virulence factor, cell wall LPS, are manifested by a massive rise in epithelial cell apoptosis, increase in proinflammatory cytokine production, and the disturbances in NO signaling pathways [21–23]. Therefore, in this study we investigated the nature of the impairment in NOS generating system induced in sublingual salivary gland acinar cells by *P. gingivalis *LPS.

Our findings revealed that the LPS-induced enhancement in the acinar cell apoptosis and the disturbances in NO were associated with the suppression of in cNOS activity and a marked upregulation in the activity of iNOS. Further, preincubation with a peptide hormone, ghrelin, recently identified in saliva and recognized for its modulatory effect on the inflammatory responses to bacterial infection [16, 17, 19], elicited a decrease in the LPS-induced apoptosis and iNOS. Moreover, ghrelin countered the LPS-induced suppression in the activity of cNOS. These results are thus in keeping with the conclusions of earlier studies demonstrating that the proapoptotic effects of *P. gingivalis *LPS are directly linked to the events of associated with iNOS induction and caspase 3 activation [16, 22]. The fact that the LPS-induced proapoptotic events were accompanied by a marked decrease in cNOS activity, while the countering effect of ghrelin was reflected in a decrease in iNOS and upregulation in cNOS, attests to the modulatory role of cNOS-derived NO on the apoptogenic signal propagation. The accumulating evidence, furthermore, suggests that ghrelin plays a major role in the regulation of local inflammatory responses through upregulation in cNOS-induced NO production [19, 20, 30] Moreover, the cNOS-derived NO has been implicated in the inhibition of apoptogenic signal through S-nitrosylation of the key executioner caspase, caspase-3 [3, 14, 16], and there are recent reports as to the regulation of cNOS activity through the enzyme protein S-nitrosylation [11, 12].

Indeed, the available literature data indicate that the activity of cNOS is regulated by a complex set of co- and posttranslational modifications, including fatty acid addition through N-myristoylation and thiopalmitoylation, interaction with regulatory cofactors, and the protein phosphorylation [7–10, 27]. Hence, to gain an insight into the mechanism of *P. gingivalis *LPS-induced changes in cNOS activity and the effect of ghrelin, we focused further on examining the events associated with cNOS activation. We found that, in keeping with the documented involvement of Src/Akt pathway in cNOS posttranslational activation through phosphorylation at Ser^1179^ [9, 10, 20, 27], the countering effect of ghrelin on the LPS-induced changes in cNOS activity as well as apoptosis were subject to suppression by Src kinase inhibitor, PP2, Akt inhibitor, SH-5, and cNOS inhibitor, L-NAME. However, preincubation with nitrosothiols reducing agent, ascorbate [25, 26], resulted in amplification of the effect of ghrelin on cNOS activity. Together, these data suggest that ghrelin countering effect on the LPS-induced proapoptotic events occurs with the involvement of Src/Akt kinase-mediated cNOS activation through phosphorylation, that appears to be dependent upon the extent of cNOS protein S-nitrosylation. Our results furthermore, are supported by the recent reports demonstrating that ascorbate treatment both increases cNOS activity and reduces the enzyme protein S-nitrosylation [11, 12]. Indeed, the growing evidence suggests that like posttranslational modification through phosphorylation, the protein S-nitrosylation is a targeted and reversible physiologically important posttranslational event that regulates protein activity during cell signaling [3–6, 11, 16, 30].

Our assertion that *P. gingivalis *LPS-induced S-nitrosylation of the acinar cell cNOS interferes with the enzyme activation through its protein phosphorylation is supported further by the results biotin switch assay. We found that the acinar cells exposed to incubation with the LPS alone showed a marked increase in cNOS S-nitrosylation. The countering effect of ghrelin on the LPS-induced suppression in cNOS activity was reflected in the loss in S-nitrosylation and the increase in the enzyme protein phosphorylation, while the suppression of ghrelin by Src kinase inhibitor, PP2, was manifested in the inhibition of cNOS phosphorylation. Interestingly, recent data indicate that stimulus-activated (VEGF) targeting of cNOS to cellular membrane is associated with both an increase in enzyme protein phosphorylation as well as a decrease in its S-nitrosylation [11, 12].

Finally, our data also revealed the dependence of cNOS S-nitrosylation on *P. gingivalis *LPS-induced iNOS activity. We observed that suppression of the LPS-induced iNOS activity with a specific inhibitor, 1400 W, led to a substantial decrease in cNOS S-nitrosylation. These results are thus indicative of the involvement of iNOS-derived NO in the suppression of cNOS activity through S-nitrosylation.

In conclusion, the data provided in our study suggest that *P. gingivalis *LPS-induced disturbances in the acinar cell NO signaling pathways result from upregulation in iNOS that leads to the induction in NO generation and cNOS S-nitrosylation that interferes with its activation. Furthermore, our findings demonstrate that following ghrelin stimulation, cNOS appears to undergo a rapid activation associated with a marked decrease in its S-nitrosylation and the increase in the protein phosphorylation.
